# Transcriptome profiling identified differentially expressed genes and pathways associated with tamoxifen resistance in human breast cancer

**DOI:** 10.18632/oncotarget.23694

**Published:** 2017-12-26

**Authors:** Xin Men, Jun Ma, Tong Wu, Junyi Pu, Shaojia Wen, Jianfeng Shen, Xun Wang, Yamin Wang, Chao Chen, Penggao Dai

**Affiliations:** ^1^ National Engineering Research Center for Miniaturized Detection Systems, College of Life Science, Northwest University, Xi'an, PR China

**Keywords:** tamoxifen, resistance, transcriptome, RNA-seq, breast cancer

## Abstract

Tamoxifen (TAM) resistance is an important clinical problem in the treatment of breast cancer. In order to identify the mechanism of TAM resistance for estrogen receptor (ER)-positive breast cancer, we screened the transcriptome using RNA-seq and compared the gene expression profiles between the MCF-7 mamma carcinoma cell line and the TAM-resistant cell line TAMR/MCF-7, 52 significant differential expression genes (DEGs) were identified including *SLIT2, ROBO, LHX, KLF*, *VEGFC, BAMBI, LAMA1, FLT4, PNMT, DHRS2, MAOA* and *ALDH.* The DEGs were annotated in the GO, COG and KEGG databases. Annotation of the function of the DEGs in the KEGG database revealed the top three pathways enriched with the most DEGs, including pathways in cancer, the PI3K-AKT pathway, and focal adhesion. Then we compared the gene expression profiles between the Clinical progressive disease (PD) and the complete response (CR) from the cancer genome altas (TCGA). 10 common DEGs were identified through combining the clinical and cellular analysis results. Protein-protein interaction network was applied to analyze the association of ER signal pathway with the 10 DEGs. 3 significant genes (*GFRA3, NPY1R* and *PTPRN2*) were closely related to ER related pathway. These significant DEGs regulated many biological activities such as cell proliferation and survival, motility and migration, and tumor cell invasion. The interactions between these DEGs and drug resistance phenomenon need to be further elucidated at a functional level in further studies. Based on our findings, we believed that these DEGs could be therapeutic targets, which can be explored to develop new treatment options.

## INTRODUCTION

Public health data indicate that breast cancer is the most frequent and the second leading cause of death due to malignant diseases among women in the world. Every year, more than 1 million women suffer from breast cancer, and more than 410000 of them lose their lives because of breast cancer [1].

For patients with ER-positive disease, adjuvant anti-estrogen treatment can significantly improve the outcome. Breast cancer mortality has decreased in recent years due to the long-term adjuvant anti-estrogen therapy clinically. As a selective estrogen-receptor modulator (SERM), TAM is approved by the Food and Drug Administration (FDA) to treat both early and advanced ER-positive breast cancer in pre- and post-menopausal women, and recommended for 10 years to reduce the incidence of breast cancer [2].

However, endocrine therapy resistance is almost inevitable in 20∼30% of all ER-positive breast tumors, which limits their available treatment options. Endocrine therapy resistance is closely associated with ER related pathways in ER-positive breast cancer, including loss of ER expression, posttranslational modifications of ER, increased AP1 activity, deregulation of ER co-activators, and deregulation of the cell cycle [3–7]. Besides, emerging evidence suggests that TAM resistance can be caused by increased receptor tyrosine kinase signaling, which leading to the activation of the Erk and PI3K pathways. Preclinical studies had also shown that breast cancer cells with activated PI3K/Akt/mTOR signaling are resistant to antiestrogen therapy [8]. Therefore, our purpose of this study was to identify the DEGs related to acquired TAM resistance using clinical and cellular RNA-seq data.

Next-generation is an increasingly attractive method for the genome-wide transcriptomic studies and allows the hypothesis-neutral investigations on the expression of both known and novel transcripts with a high resolution. TCGA provides a unique opportunity to examine breast cancer on a large scale, both at a clinical and molecular level, since it contains expression data from over 500 cases. RNA-Seq (IlluminaGA_RNASeqV2 platform) for breast cancer samples can be downloaded from TCGA. In order to understand drug resistance *in vitro*, we established the models of drug-resistant cell lines. Furthermore, such cell lines can be used to search for prognostic or predictive biomarkers and identify potential targets for therapy [9].

**Figure 1:**
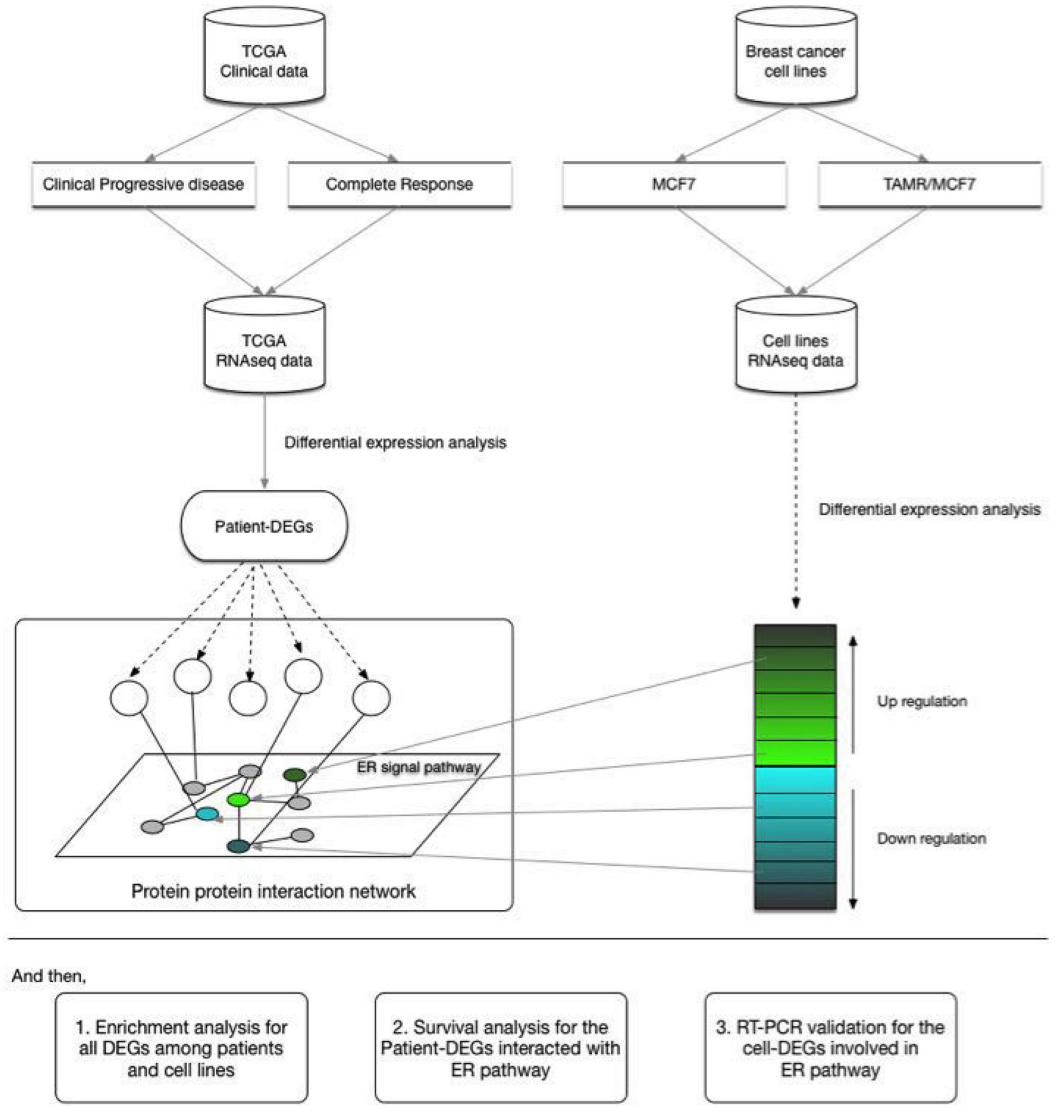
Analysis design of the study The study was designed to screen transcriptome in clinical and cellular levels of breast cancer. The DEGs were identified in two groups. PPI was performed to analyze the association between the common DEGs and ER related pathway. And then, Enrichment analysis was conducted to understand the function of DEGs; Survival analysis was conducted to find the association of DEGs with ER signal pathway; qRT-PCR was performed to validate RNAseq results.

In this study, we screened transcriptome of MCF-7 and TAMR/MCF-7 cell lines. Then we compared the gene expression profiles between the PD and the CR samples from TCGA. Through comparing the cellular and clinical data, several significant DEGs were identified. We explored the interaction between the common DEGs with ER signal pathway by the protein-protein interaction network. And then, we analyzed the association between the DEGs with DFS with patients. At last, the RNA-seq results were verified by quantitative RT-PCR (qRT-PCR). These findings may improve understanding TAM resistance in breast cancer patients, and also provide potential markers for prognosis and treatment. The flamework of this research is showed in Figure 1.

## RESULTS

### Validation of drug inhibition in MCF-7 and TAMR/MCF-7 cells

In order to understand TAM resistance *in vitro*, cell models were established by continuous exposure to a certain concentration of 4OH-TAM for 6 months. The clones of single cell were derived from TAMR/MCF-7 cells by a limiting dilution strategy.

**Figure 2:**
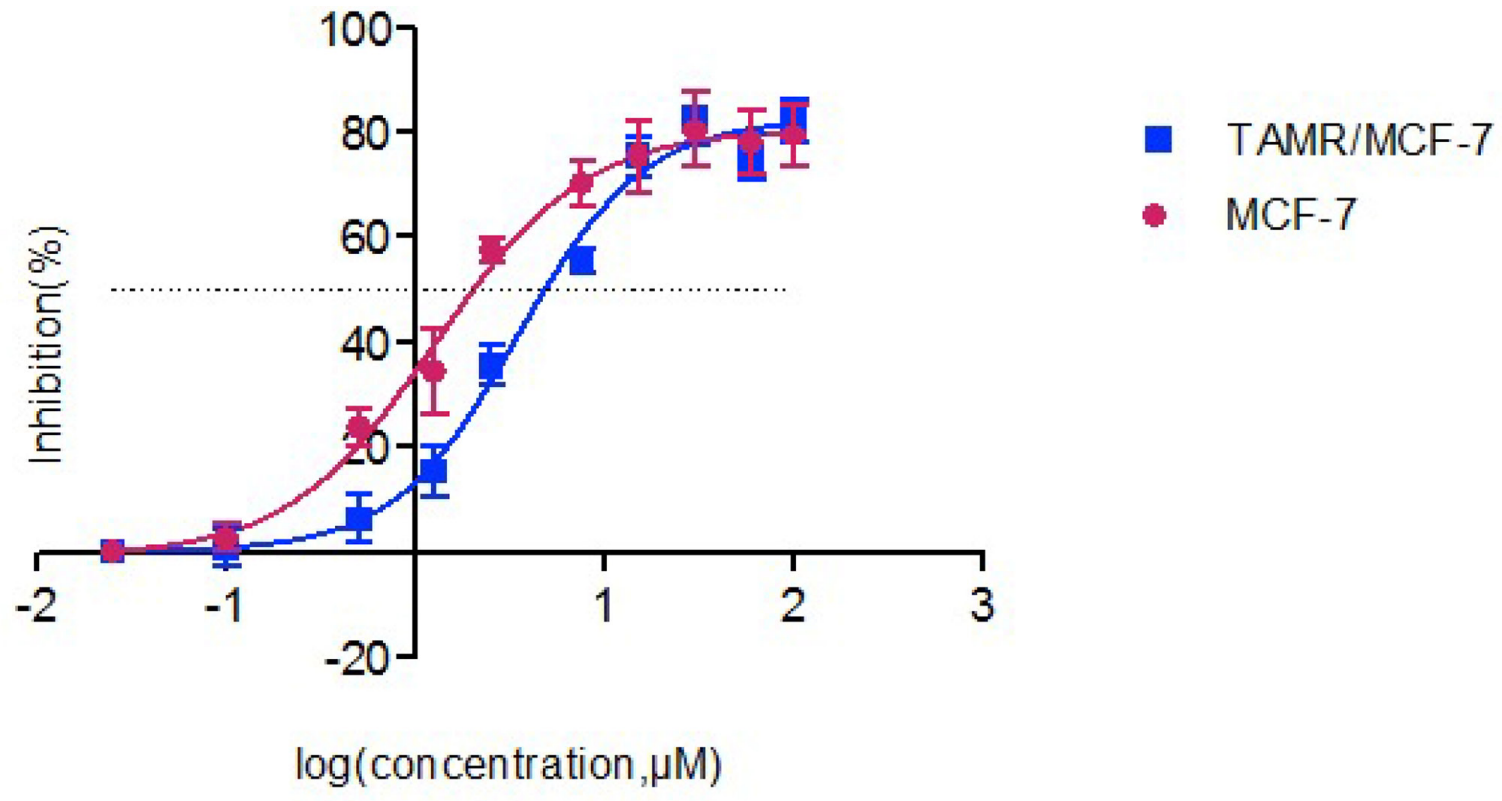
The inhibitory effects of different concentrations of 4-OH TAM on MCF-7 and TAMR/MCF-7 cells As assessed by the CCK-8 assay, cell viability of MCF-7 and TAMR/MCF-7 was determined after exposure to increasing amounts of 4-OH TAM for 48h. Results represent the average of triplicate wells and are representative of three independent experiments. Red bars and symbols, MCF-7; blue bars and symbols, TAMR/MCF-7.

We tested the cytotoxicity of 4OH-TAM in MCF-7 and TAMR/MCF-7 cells. The fifty percent of inhibitory concentration value (IC50) (mean ± SD) of 4OH-TAM in the MCF-7 and TAMR/MCF-7 cells is shown in Table 1. Figure 2 shows the dose-response growth inhibitory curve. Cytotoxicity tests showed that the tolerance of cells to the 4-OH TAM (endoxifen) of IC50 is higher than that of MCF-7, and the resistance index is 2.82, which indicates that we successfully established a 4-OH TAM resistant cell model. The resistance to 4-OH TAM in the model is adaptive.

**Table 1:** The 50% inhibitory concentration value (mean ± SD) of TAM in MCF-7 and TAMR/MCF-7 cells

Samples	Treat with TAM	
	IC50^*^	RI^**^
MCF-7	1.241±0.18	/
TAMR/MCF-7	3.505±0.29	2.82

^*^The 50% inhibitory concentration value.

^**^Resistant index.

### Transcriptome sequencing analyses

**Figure 3:**
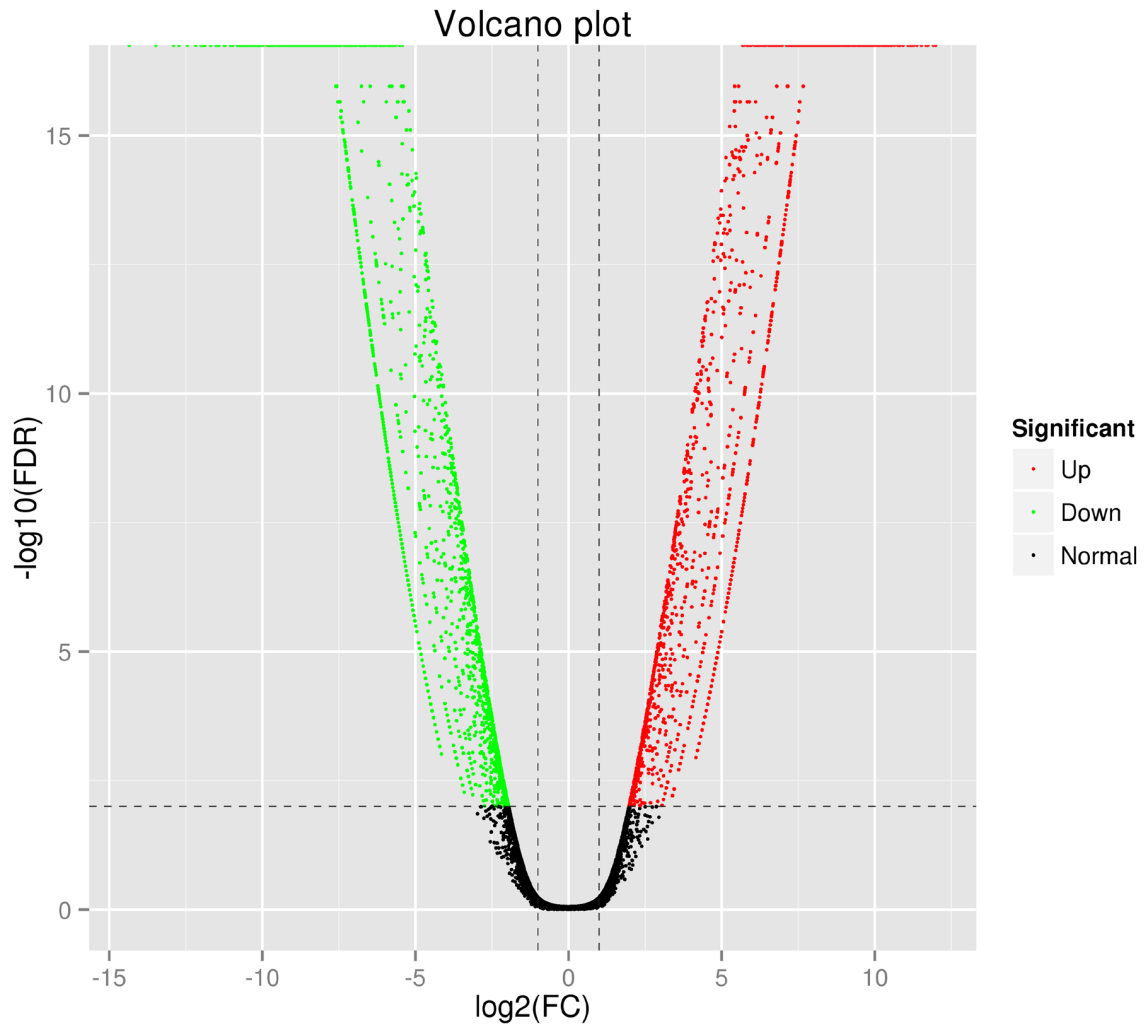
Distribution of the differentially expressed genes shown as a volcano plot Each point represents a gene. The green dots represent the down-regulated differentially expressed genes, red dots represent the up-regulated differentially expressed genes, and black dots represent non-differentially expressed genes.

After trimming and removing low quality reads, we acquired 22.24 Gb of clean data. The Q30 percentage was 90.94%. Table 2 shows the statistical summary of transcriptome sequencing. We took human genome GRCh37 as reference to align the reads by using the TopHat software. 84.72% and 85.58% reads were mapped to the reference for the MCF-7 cells and TAMR/MCF-7 cells respectively. The statistical summary of the mapping results is shown in Table 3. The RSEM package was used to normalize transcript abundances. The FPKM were estimated with the selection criteria of q value < 0.005 and |log2 (fold change)| > 2. We identified a total of 3276 DEGs between the MCF-7 and TAMR/MCF-7 cells. Among all the DEGs, 1449 up-regulated and 1827 down-regulated DEGs were identified. Figure 3 presents the Volcano Plot to examine the difference in the expression level of genes in two group of samples and the statistical significance of the differences.

**Table 2:** Statistical summary of transcriptome sequencing

Samples	MCF-7	TAMR/MCF-7
**Raw reads**	33,203,223	34,449,037
**clean reads**	30,131,447	31,371,798
**GC Content**	52.76%	52.96%
**%≥Q30**	90.94%	90.79%

**Table 3:** Statistical summary of the mapping results

Samples	MCF-7	TAMR/MCF-7
**Total Reads**	60,262,894	62,743,596
**Mapped Reads**	53,925,432(89.48%)	55,975,916(89.21%)
**Uniq Mapped Reads**	51.054.181(84.72%)	53.697.187(85.58%)
**Multiple Map Reads**	2.871.251(4.76%)	2,278,729(3.63%)
**Reads Map to ‘+’**	26,774,826(44.43%)	27,834,465(44.36%)
**Reads Map to ‘-’**	26,821,128(44.51%)	27,845,540(44.38%)

### DEG annotation and enrichment

**Figure 4:**
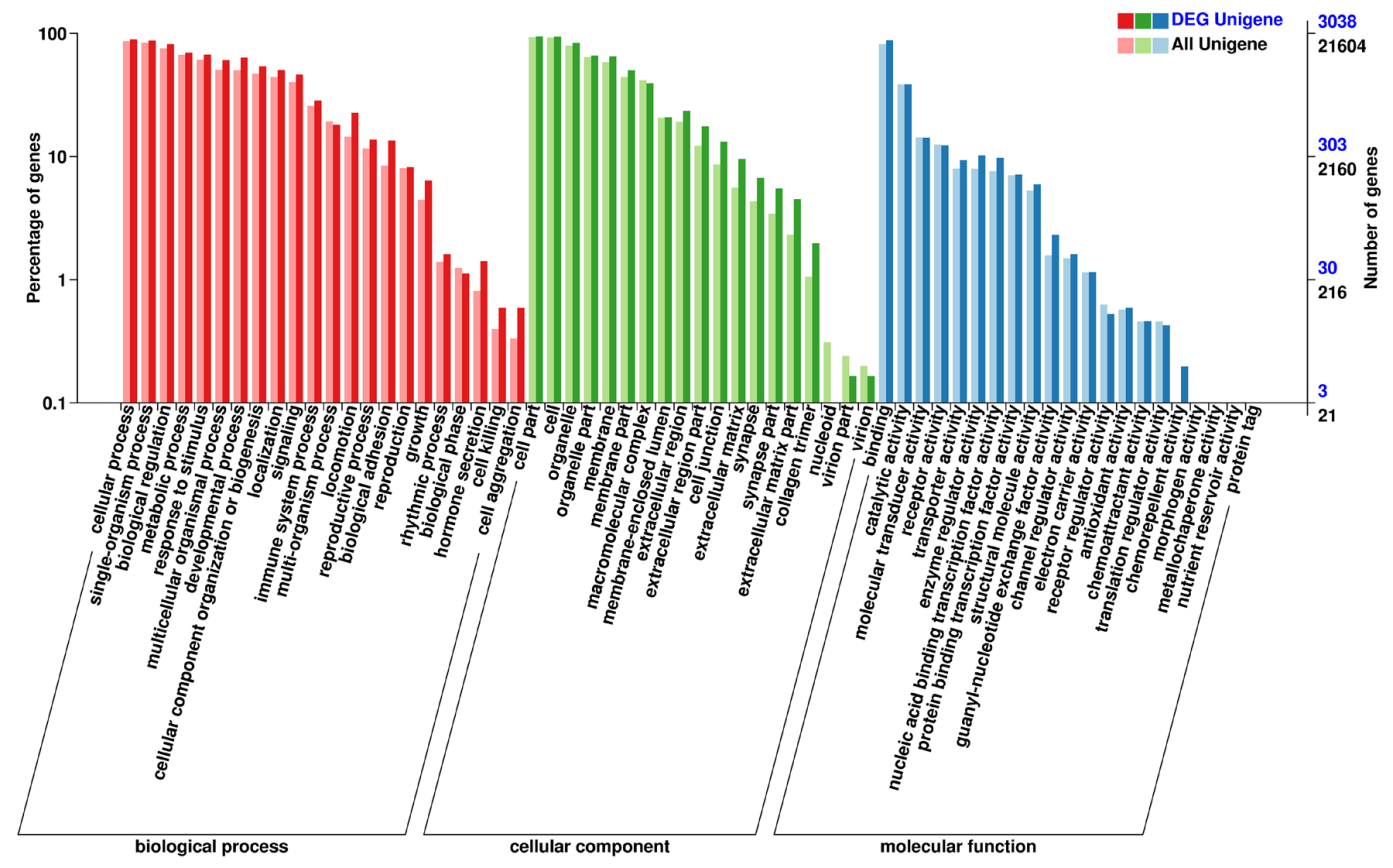
GO enrichment analysis of the DEGs The horizontal axis shows the secondary nodes of three categories in GO. The vertical axis displays the percentage of annotated genes versus the total gene number. The dark color columns display annotation information of the total genes and the light color columns represent annotation information of the differentially expressed genes only.

The DEGs were annotated in the GO, COG, and KEGG database respectively. Gene Ontology (GO) enrichment analysis of the DEGs was implemented using the GOseq R packages based on Wallenius non-central hyper-geometric distribution [10]. Figure 4 shows the results of DEGs annotated in the GO database. In the category of cellular component, the highest proportion of DEGs was distributed in the cell or cellular part sub-category (2983 DEGs, representing 98.19% of all DEGs). In the category of molecular function, the highest proportion of DEGs was distributed in the binding sub-categories (2836 genes, representing 93.35% of all DEGs). In the category of biological processes, the highest proportion of DEGs was distributed in cellular processes (2885 genes, representing 94.96% of all DEGs).

**Figure 5:**
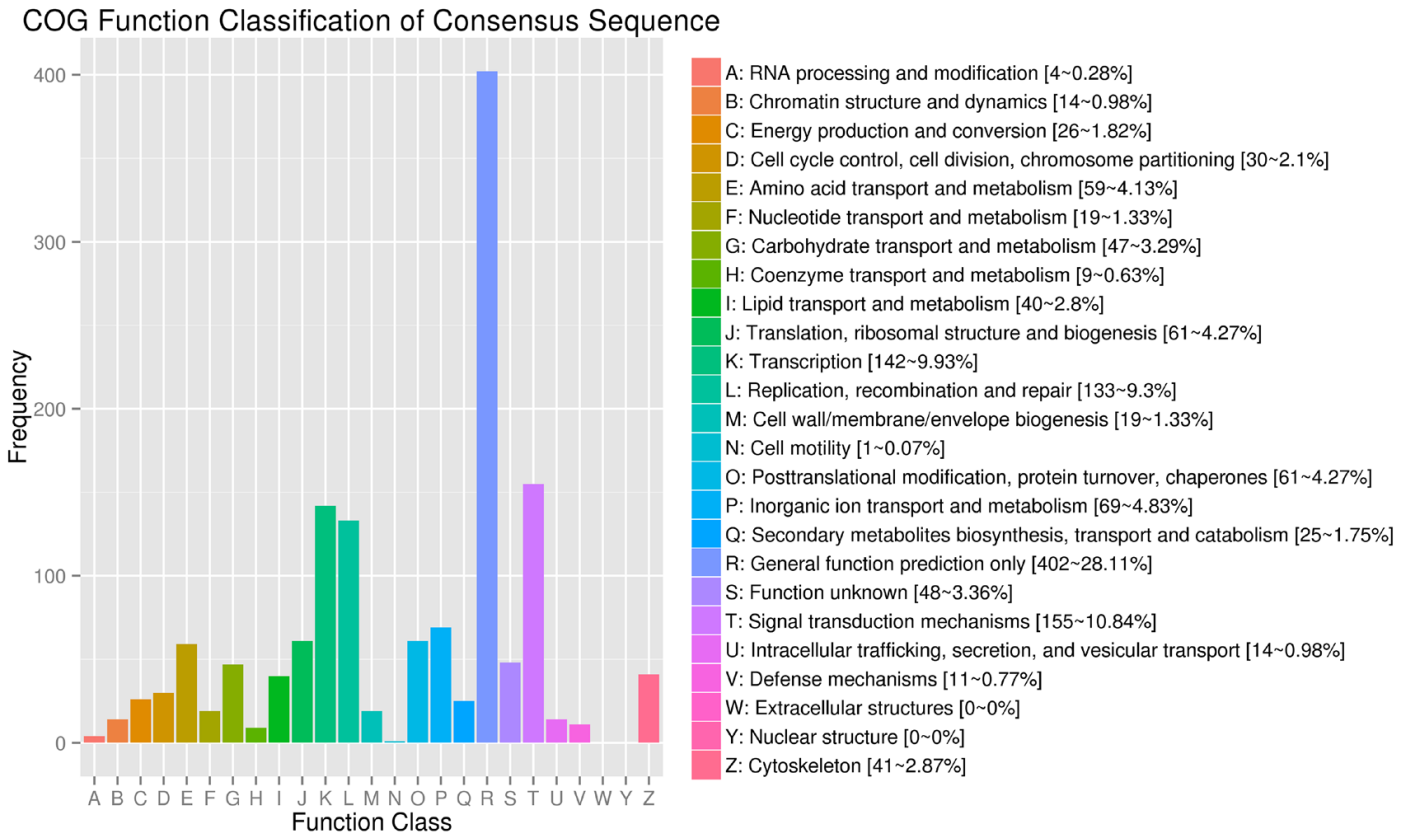
COG function classification of the annotated DEGs 938 annotated DEGs are distributed into 25 COG functional categories. The COG categories are shown on the horizontal axis and gene numbers and proportions are plotted on the vertical axis. COG function classification of the annotated DEGs. 938 annotated DEGs are distributed into 25 COG functional categories. The COG categories are shown on the horizontal axis and gene numbers and proportions are plotted on the vertical axis.

COG built on coding proteins is an orthologous gene product database. Figure 5 shows that 938 annotated DEGs are distributed into 25 COG functional categories in our study. Among all the enriched DEGs in COG, 402 genes are distributed into the category of general function prediction only, representing 28.11% of all genes; 155 genes are distributed into the category of signal transduction metabolism, representing 10.84% of all genes; 142 genes are distributed into the category of transcription, representing 9.93% of all genes; 133 genes are distributed into the category of replication, recombination, and repair, representing 9.3% of all genes, and 69 genes are distributed into the category of Inorganic ion transport and metabolism, representing 4.83% of all genes.

**Figure 6:**
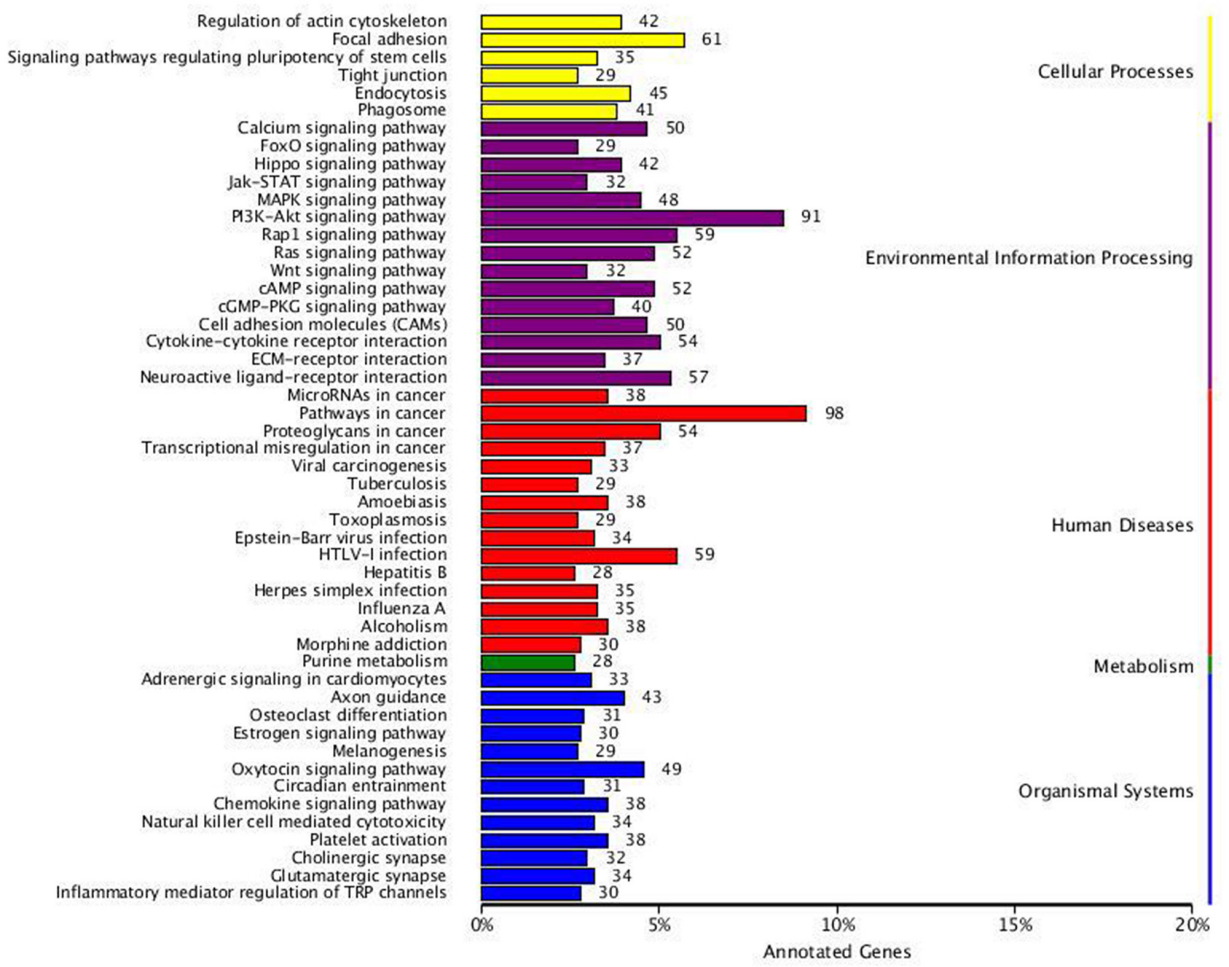
KEGG categories of differentially expressed genes The vertical axis lists the names of the metabolic pathways in the KEGG database, and the horizontal axis shows the proportion of annotated genes in each pathway versus the total number of annotated genes.

In the present study, the KOBAS [11] software was used to test the statistical enrichment of DEGs in KEGG pathways. We distributed a total of 2005 DEGs into 278 pathways in the KEGG database (http://www.genome.jp/kegg/) [12]. The top 50 DEGs-enriched pathways are provided in Figure 6. The DEGs-enriched pathways included pathway in cancer which largest number of DEGs involved in, cellular processes such as focal adhesion, regulation of actin cytoskeleton, and environmental information processing such as the mitogen-activated protein kinase (MAPK), Rap1, PI3K-AKT, Ras and CAM (cell adhesion molecules) signaling pathway. Tables 4 and 5 provide 52 significant DEGs identified to regulate and participate in many biological processes including tumor cell migration and invasion, cell proliferation and survival.

**Table 4:** The regulated trend and log2FC of identified DEGs

Gene	ID	log2FC	Regulated
ESR1	ENSG00000091831	-10.51	down
IGFBP5	ENSG00000115461	-9.492	down
ADAMTS9	ENSG00000163638	-7.926	down
ALDH3B2	ENSG00000132746	-7.506	down
RTN4RL1	ENSG00000185924	-7.329	down
MYH10	ENSG00000133026	-6.664	down
EDN1	ENSG00000078401	-3.940	down
HGFAC	ENSG00000109758	-3.885	down
DHRS2	ENSG00000100867	-3.664	down
PDGFA	ENSG00000197461	-3.444	down
MAOA	ENSG00000189221	-3.248	down
FLT4	ENSG00000037280	-3.126	down
INS-IGF2	ENSG00000129965	-2.575	down
BAMBI	ENSG00000095739	-2.363	down
E2F8	ENSG00000129173	-2.202	down
ALDH5A1	ENSG00000112294	-2.264	down
ALDH6A1	ENSG00000119711	-2.348	down
ALDH4A1	ENSG00000159423	-2.495	down
CDKN2A	ENSG00000147889	9.0156	up
LHX9	ENSG00000143355	6.627	up
CCNA1	ENSG00000133101	6.850	up
COL8A1	ENSG00000144810	5.816	up
ALDH3A1	ENSG00000108602	5.584	up
LAMA1	ENSG00000101680	5.532	up
CDKN1C	ENSG00000129757	4.992	up
KLF12	ENSG00000118922	4.708	up
CDK14	ENSG00000058091	4.653	up
PNMT	ENSG00000141744	4.488	up
VEGFC	ENSG00000150630	3.953	up
EDNRA	ENSG00000151617	3.844	up
COL9A3	ENSG00000092758	3.420	up
ALDH1L2	ENSG00000136010	3.301	up
CDK6	ENSG00000105810	3.214	up
TIMP2	ENSG00000035862	2.719	up
ROBO1	ENSG00000169855	3.219	up
KLF7	ENSG00000118263	2.946	up
KLF13	ENSG00000169926	2.432	up
COL4A5	ENSG00000188153	2.122	up
SKP2	ENSG00000145604	2.527	up
SLIT2	ENSG00000145147	2.304	up
CDKN2C	ENSG00000123080	2.234	up

**Table 5:** The regulated trend and log2FC of 10 common identified DEGs from TCGA and cell lines

Gene	#ID	Clinical data		Cellular data	
		log2FC	Regulated	log2FC	Regulated
NPY1R	ENSG00000164128	-3.744	down	-10.78	down
C2CD4D	ENSG00000225556	-3.011	down	-7.062	down
SOX8	ENSG00000005513	-2.879	down	-4.889	down
GABRP	ENSG00000094755	-4.146	down	-4.628	down
GFRA3	ENSG00000146013	-3.731	down	-4.587	down
PTPRN2	ENSG00000155093	-2.437	down	-4.409	down
ARNT2	ENSG00000172379	-2.834	down	-2.711	down
ATP6V1C2	ENSG00000143882	-2.831	down	-2.411	down
GRB14	ENSG00000115290	-4.899	down	-2.126	down
NELL2	ENSG00000184613	-4.134	down	4.269	up

SLIT, characterized as a member of axon guidance molecules (AGMs) plays an important role in the mammary gland to maintain proliferation and adhesion of normal cell during development. Previous study has shown that SLIT plays a crucial role in breast cancer as a tumor suppressor and oncogene; lower expression of SLIT is associated with tumorigenesis and tumor progression [13]. Our results in Table 4 show that SLIT2 and ROBO are increasingly expressed in TAMR/MCF-7 cells than in MCF-7 cells. It suggested that SLIT might participate in regulating proliferation during breast cancer resistance. The secreted glycoproteins encoded by *SLIT2* are ligands for the ROBO family of immunoglobulin receptors [14]. These data presented above suggest that *SLIT2* and *ROBO* are associated with TAM resistance and progressive disease of breast cancer. Therefore, we believe that these two genes could be targets for overcoming TAM resistance and developing more effective therapeutic strategies in breast cancer.

By comparing the TAMR/MCF-7 cells with the parental cells, transcriptional regulators encoded by *LHX* and *KLF*, growth factors encoded by *VEGFC, IGF, AGT, TGFA, and HGFAC*, cytokines encoded by *EDN1* and *TIMP2*, G-protein coupled receptor encoded by *EDNRA,* and transmembrane receptor encoded by *BAMBI,* enzymes encoded by *MYH10* and *FLT4,* and other genes including *LAMA1* and *COL,* were found to be differentially expressed in our study. Previous studies have shown that the Kruppel-like family of Transcriptional regulators, encoded by *KLF* genes, regulates not only physiological processes but also the pathogenesis of many diseases, including cancer and inflammatory disorders [15, 16]. *KLF7, KLF12* and *KLF13* were found to be up-regulated in our trascriptome analysis results. LAMA1, an extracellular glycoprotein of the laminin family, is an essential component of basement membranes, being involved in tumorigenesis and other biological processes [17]. *VEGFC* results in the promotion of angiogenesis and/or lymphangiogenesis [18]. Moreover, it has been reported that FLT4 significantly correlate with the different stages of cervical carcinogenesis [19]; the ligand of FLT4 might effecting tumor cells directly to affect cancer development and progression [20].

Moreover, in our study, members of the pathway “Noradrenaline and Adrenaline Degradation” encoded by *PNMT, DHRS2, MAOA,* and *ALDH* were found to be differentially expressed in the TAMR/MCF-7 cells. The protein product of *PNMT* plays a crucial role in regulating epinephrine production. *DHRS2* is reported to regulate the biological process in the p53 pathway [21, 22]. In our study, we showed that *DHRS2* was down-regulated in the TAMR/MCF-7 cells, which suggests that decreased expression of *DHRS2* is associate with tumor progression, wherein it acts by inhibiting p53 protein expression. Breast cancer cells are characterized by high ALDH activity and ALDH activity is associated with up-regulated proliferation and invasion [23]. In our study, *ALDH3A1* and *ALDH1L2* were found to be up-regulated, while *ALDH3B2, ALDH5A1, ALDH6A1,* and *ALDH4A1* were found to be down-regulated. More research is necessary to elucidate the potential association between acquired TAM resistance and *ALDH*.

### DEGs expression in response to TAM in breast cancer patients

**Figure 7:**
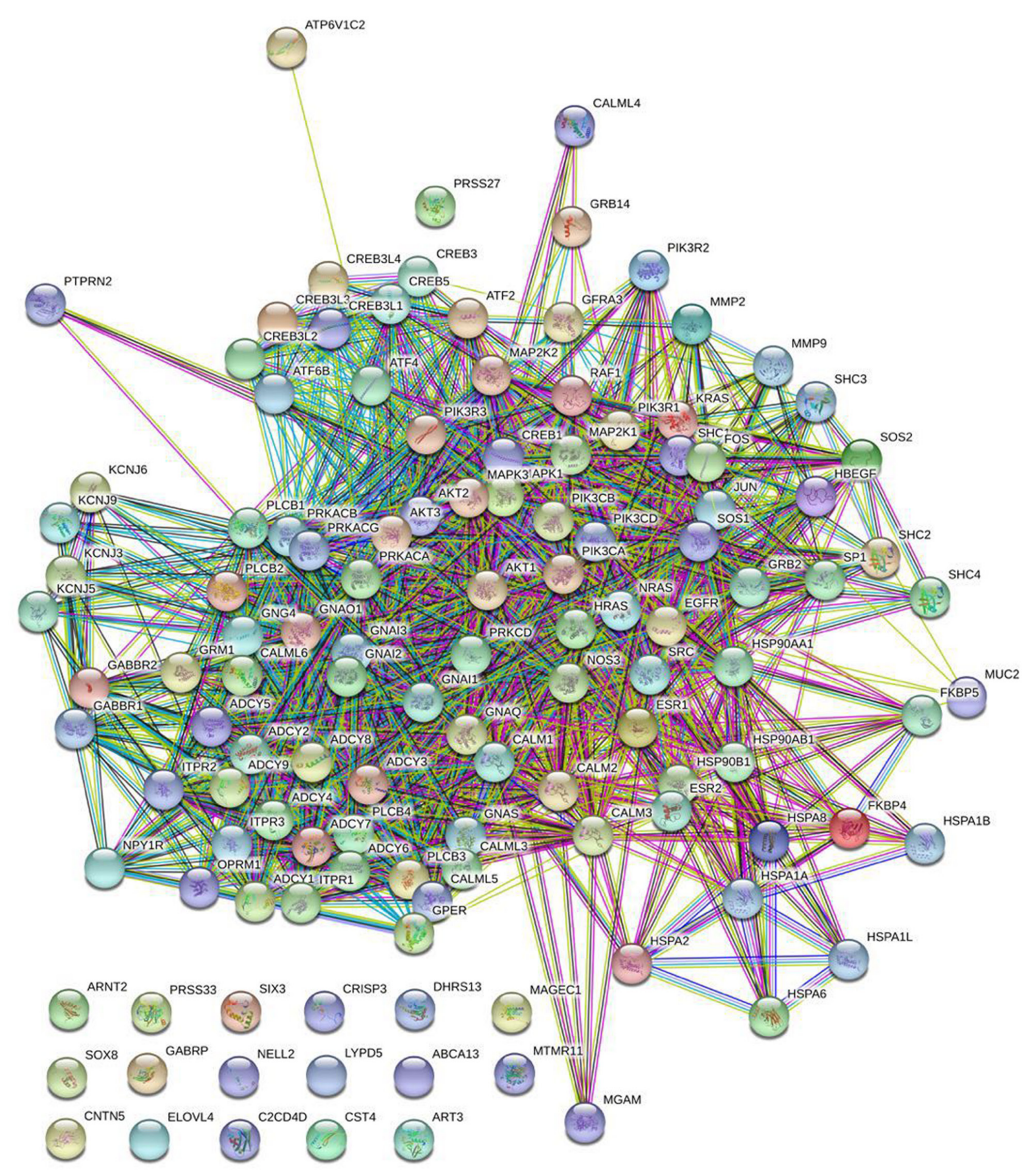
Protein-protein interaction network analysis DEGs (n=26) of patients and genes(n=98) involved in ER signal pathway were integrated using STRING website to explore the association between these DEGs with ER functional related genes. The result showed that three common DEGs including GFRA3, NPY1R and PTPRN2 were closely related to ER related pathway.

To predict the DEGs related to TAM resistance in breast cancer patients after treatment with TAM, we examined gene expression data using TCGA RNA-seq dataset from 22 unique breast cancer samples. This dataset has both molecular and clinical information. Among 26 DEGs that were identified in PD compared with CR samples, 10 common genes showed in Table 5 are also differentially expressed in MCF-7 and TAMR/MCF-7 cell lines. These 10 DEGs were all down-regulated in PD samples. The results were consistent with cellular data except for NULL which was up-regulated in TAMR/MCF-7. Protein-protein interaction network analysis was conducted to analyze the association between the 10 common DEGs and genes involved in ER related pathway. The result showed in Figure 7 reveal that GFRA3, NPY1R and PTPRN2 are closely related to ER related pathway. These genes were reported to be associated with tumorigenesis and tumor progression in previous study.

Eftang LL et al. reported that the *GFRA3* promoter region was found to be hypermethylatied in almost all tumors, and its correlation with survival and other clinicopathological parameters may have important prognostic significance [24]. *NPY1R* was found to participate in the inhibition of cell proliferation via inactivating mitogen-activated protein kinase signal pathway in HCC cells and play an inhibitory role in tumor growth [25, 26]. *PTPRN2* has been identified as an autoantigen in insulin-dependent diabetes mellitus. Loss of *PTPRN2* in breast cancer cells promoted apoptosis and blocked tumor formation in mice, whereas enforced expression of PTPRN2 in nontransformed human mammary epithelial cells exerted a converse effect. It was reported that *PTPRN2* was a novel candidate biomarker and therapeutic target in breast cancer [27].

Previous study suggested that *GABRP* is differentially expressed in breast cancer. With the progression of breast cancer, *GABRP* was down-regulated progressively. It may be a useful marker in prognosis of breast cancer [28].

### qRT-PCR verification

**Figure 8:**
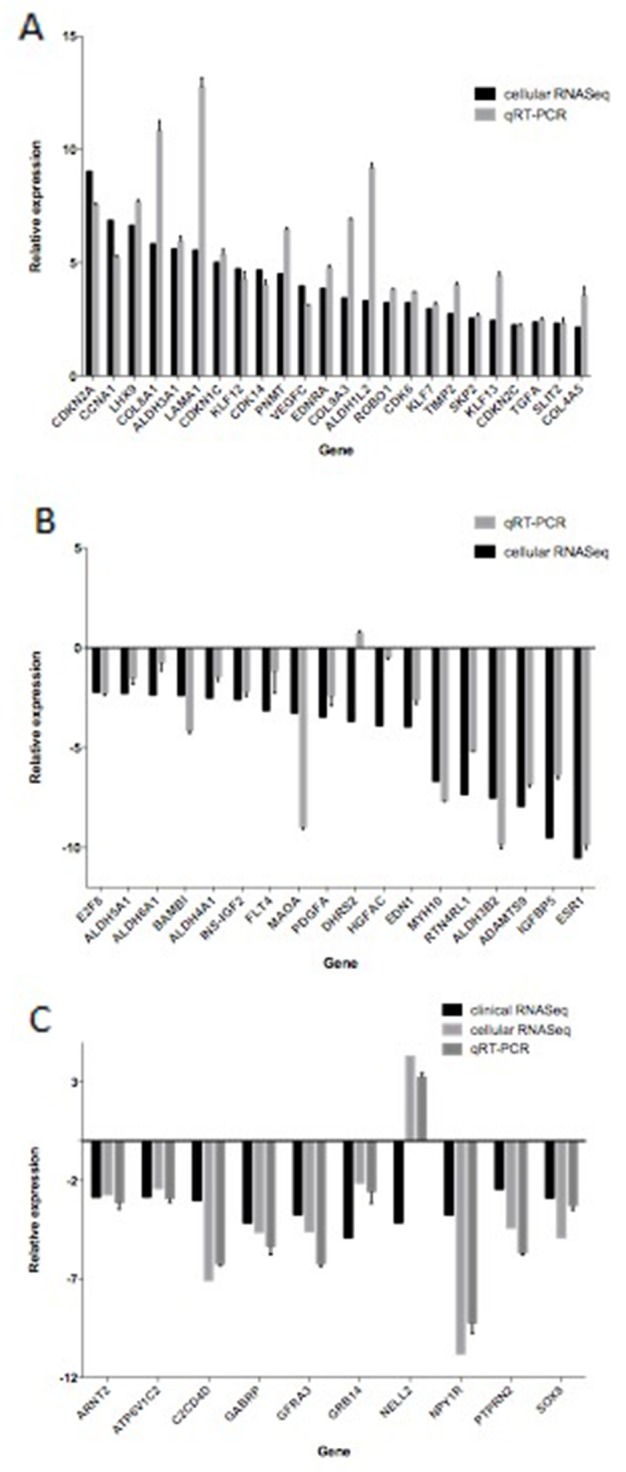
The differentially expressed genes detected by transcriptome sequencing confirmed by qRT-PCR qRT-PCR was performed for 52 genes that were identified to be differentially expressed between MCF-7 and TAMR/MCF-7 cells. The expression level of each gene was normalized to the level in MCF-7 cells. A and B showed relative expression levels of 18 down regulated and 24 up regulated DEGs respectively. C showed relative expression levels of 10 common DEGs identified in clinical data and cell lines.

In order to verify the transcriptome sequencing results, we examined the expression of 52 significant DEGs in the MCF-7 and TAMR/MCF-7 cells by qRT-PCR. Figure 8 shows a summary of the transcriptome sequencing and qRT-PCR results. The relative expression was characterized by log_2_FC and ∆∆Ct. Although the relative expression of 52 DEGs was not in the same levels in qRT-PCR and RNA-Seq, the regulated trends of 51 DEGs were entirely consistent except for *DHRS2*. It was worth noting that 9 of 10 down-regulated DEGs identified from clinical data in PD samples were also less expressed in TAMR/MCF-7 cell line, while only the expression of *NELL2* was increased.

### Survival analysis

**Figure 9:**
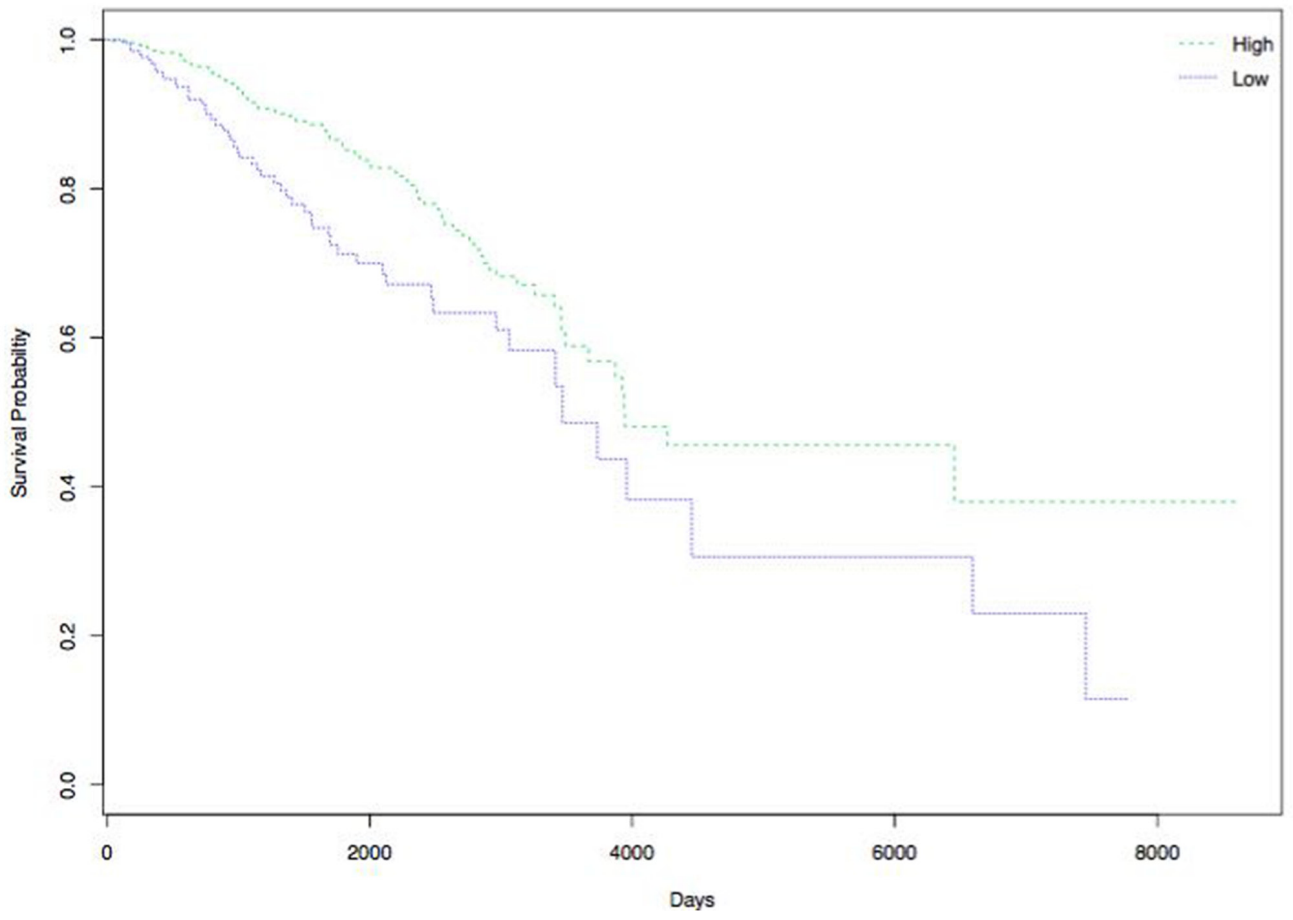
Correlation of NPY1R expression and DFS of patients with breast cancer High expression of NPY1R is associated with improved DFS. Green and blue lines indicated low and high expression groups, respectively. P <0.05 was considered to be statistically significant.

Survival analysis was performed to explore the correlation between the 4 DEGs (*GFRA3, NPY1R, PTPRN2* and *GABRP)* with patients’ survival in over 400 breast cancer samples from TCGA database. Figure 9 shows that increasingly expressed NPY1R is closely related to improved DFS. The results did not show that the other three genes were associated with DFS.

## DISCUSSION

Development of TAM resistance is a severe problem in breast cancer therapy. To overcome TAM resistance, understanding the underlying mechanisms of the resistance is essential. Our purpose of this study was to identify the DEGs associated with TAM resistance. First, we established TAMR/MCF-7 cell line and tried to determine the gene expression profiles of TAMR/MCF-7 and MCF-7 cells, using next-generation sequencing (NGS) technique to screen DEGs. Finally, 52 significant DEGs were identified through screening the gene expression profiles by RNA-Seq. These DEGs encode transcriptional regulators, growth factors, cytokines, G-protein coupled receptors, transmembrane receptors and enzymes which are crucial in significant DEGs enriched pathways to regulate biological processes especially cancer development and tumor progression.

For further study, 22 breast cancer patients treated with TAM were chosen from TCGA and grouped into Complete Response (CR) and Clinical Progressive Disease (PD) groups. On the basis of RECIST, changes in tumor volume are used to measure the effect of clinical treatment [29]. We considered the CR samples (n=18) as the TAM sensitive group and the PD samples (n=4) as the TAM resistance group. We analyzed RNA-Seq data of these two sets. 26 significant DEGs were identified in PD vs. CR.

We compared the transcriptome analysis results of clinical and cellular RNA-seq data. 10 common DEGs were identified. These 10 DEGs were down-regulated in PD samples. The results were consistent with cellular data except for *NULL* that was up-regulated in TAMR/MCF-7 cells. These data suggest that while the cell lines of MCF-7 and TAMR/MCF-7 can be used to study TAM cytotoxicity *in vitro*, individual difference of patients and the tumor environment *in vivo* also plays a vital role in TAM cytotoxicity. Another problem in our study is that breast cancer samples data obtained from TCGA in our study are limited. Therefore, it is necessary to combine the cellular and clinical RNA-Seq analysis results and conduct a thorough investigation. The uncommon DEGs identified in cellular analysis should also be focused. The most important aspect is to elucidate the interactions between DEGs and drug-resistance phenomenon at a functional level in further study. In this study, we identify several vital genes through globally screening clinical and cellular transcriptome profiles, provide the points of penetration for further research.

As we known that TAM is an estrogen receptor antagonist used to prevent recurrence of breast cancer, so we try to analyze the relationship between these 10 DEGs and ER related pathway. Protein-protein interaction network analysis showed that *GFRA3*, *NPY1R* and *PTPRN2* were closely related to ER related pathway. These genes were reported to be associated with tumorgenesis and tumor progression in previous study. It is then proposed that these 3 DEGs have a closer association with disease progression in breast cancer. According to the cellular analysis, the results showed that ER was decreasingly expressed in TAMR/MCF-7 cells. The genes involved in ER related pathway such as *SKP2*, *CCNA1*, *E2F8*, *CDKN1C*, *CDKN2C*, *CDKN2A*, *CDK6* and *CDK14* were also differentially expressed in TAMR/MCF-7 cells. This is consistent to precious study [30]. The mechanism of Tam resistance has been explored in previous investigations from large microarray [31] and shRNA [32] screening. These studies have identified several DEGs related to ER pathway, some of which is confirmed by our study.

Kathryn J. H. et al [30] identified 1215 mRNA and 513 small RNA transcripts differentially expressed in cellular level by comparing the transcriptomes of Tam-sensitive and Tam-resistant breast cancer cells. In our study, we combined the transcriptome profiles of cell lines with RNA-seq data from TCGA and identified 52 siginificant DEGs involved in ER function and many biological processes including cell proliferation, apoptosis and survival, tumor cell migration and invasion.

In order to validate the RNA-seq results, qRT-PCR was performed to verify the expression of 52 DEGs in the other two monoclonal cell lines with the higher drug resistance index. The assay was performed for three times at least and three duplication for each time. The results showed that 52 genes expression in three cell lines were in the same level and comparatively accordant with the RNA-seq results.

In conclusion, through analyzing breast cancer RNA-seq data from TCGA and screening the transcriptome of TAMR/MCF-7 and the parental cell line, 52 DEGs are identified to be associated with TAM resistance in breast cancer. They are involved in the pathways to regulate many biological processes including cell proliferation, apoptosis and survival, tumor cell migration and invasion. 10 common DEGs are found both in cellular and clinical analysis results. 3 of them including *GFRA3*, *NPY1R* and *PTPRN2* are identified to be closely associated with ER related pathway. Studying the relationships among these DEGs may help unravel the potential mechanism of TAM resistance and disease progression in breast cancer. We suggest that these DEGs are potential biomarkers and therapeutic targets for TAM resistance in breast cancer. Using these DEGs as therapeutic targets may help develop a new treatment option for breast cancer and to predict and overcome TAM resistance.

## MATERIALS AND METHODS

### Cell culture and establishment of the TAMR/MCF-7 cell line

The wild-type human ER expression breast cancer cell line, MCF-7, was purchased from American Type Culture Collection and the cells were cultured in Dulbecco’s modified Eagle medium (DMEM) containing 10% FBS without penicillin-streptomycin. The TAMR/MCF-7 cell line was derived from the MCF-7 cell line by continuous exposure to 1μM 4OH-TAM diluted in 1% ethanol, for 6 months. The cells were maintained at 37°C in a humidified atmosphere of 5% CO_2_. The culture medium was replaced every other day. The medium for the matched control cells contained 0.1% ethanol. 4OH-TAM was purchased from Sigma and stored as aliquots at -20°C.

### Cell viability assay

The MCF-7 and TAMR/ MCF-7 cells were plated in 96-well plates (1^*^10^4^) and cultured in the medium containing 0-100 μM 4OH-TAM at 37°C for 48 h in a 5% CO_2_ humidified incubator. The CCK-8 solution (10 μL) was then added to each well, and the microtiter plate was incubated at 37°C for 4 h in the incubator. The absorbance was then measured at 450nm using Spectra MAX340 (Molecular devices, CA, USA)

### RNA preparation

Total RNA was extracted the TAMR/MCF-7 and MCF-7 cells, grown under preferred culture conditions as described above, by using TRIZOL Reagent (Invitrogen, Carlsbad, CA, USA) from. RNA was extracted and isolated as recommended by the manufacture. The concentration of total RNA was measured using the Nanodrop spectrophotometer, and integrity was assessed using the RNA Nano 6000 Assay Kit with the Agilent Bioanalyzer 2100 system (Agilent Technologies, Santa Clara, CA, USA).

### Library preparation and transcriptome sequencing

RNA libraries were prepared according to the NEB NextUltra™ RNA Library Prep Kit for Illumina (New England Biolabs, Ipswich, MA, USA) guide as per the manufacture’s recommended protocol (Illumina, San Diego, CA).

A total of 3 ng RNA per sample was used as input material for the RNA sample preparations. In brief, mRNA was purified from total RNA using poly-T oligo-attached magnetic beads. Fragmentation was carried out using divalent cations under elevated temperature in NEBNext First Strand Synthesis Reaction Buffer (5×). First-strand cDNA was synthesized using random hexamer primers and M-MuLV Reverse Transcriptase (RNase H-). Second-strand cDNA synthesis was subsequently performed using DNA Polymerase RNase H. Remaining overhangs were converted into blunt ends using exonuclease/polymerase activities. After adenylation of the 3’ ends of the DNA fragments, the NEBNext Adaptor with a hairpin loop structure was ligated to prepare for hybridization. In order to select cDNA fragments of preferentially 150–200 bp length, the library fragments were purified with the AMPure XP system (Beckman Coulter, Beverly, MA, USA). Then, 3 μL of USER Enzyme (New England Biolabs) was used with size-selected, adaptor-ligated cDNA at 37°C for 15 min, followed by 5 min at 95°C before polymerase chain reaction (PCR). PCR was performed using Phusion High-Fidelity DNA polymerase, Universal PCR primers, and Index (X) Primer. Finally, the PCR products were purified (AMPure XP system), and the library quality was assessed on the Agilent Bioanalyzer 2100 system. The clustering of the index-coded samples was performed on a cBot Cluster Generation System using TruSeq PE Cluster Kit v3-cBot-HS (Illumina), according to the manufacturer’s instructions. After cluster generation, the library preparations were sequenced on an IlluminaHiSeq 2000 platform and paired-end reads were generated.

### Gene expression and transcriptome analysis

Transcriptome analysis experiments can characterize all transcriptional activity (coding and non-coding), focus on a subset of relevant target genes and transcripts, or profile thousands of genes at once to create a global picture of cell function.

Gene expression of the TAMR/MCF-7 and MCF-7 cells was measured using the RSEM package [33] for each sample. Clean data were mapped back onto the assembled transcriptome, and the read count for each gene was obtained from the mapping results. Differential expression analysis of two samples was performed using the DEGseq R package. The *p* value was adjusted using the q value. A q value < 0.005 and |log2 (fold change)| >1 was set as the threshold for significantly differential expression.

### Function annotation of DEGs

The databases used to annotate the function of the identified DEGs included Clusters of Orthologous Groups (COG), Gene Ontology (GO), and Kyoto Encyclopedia of Genes and Genomes (KEGG). The query unigene sequences were then matched with the subject sequences in the multiple databases using BLAST (BLASTX tool for proteins and BLASTN tool for nucleotides) at an E-value cut-off of e-5 (<0.00001). GO enrichment analysis of the DEGs was implemented using the GOseq R packages based on the Wallenius non-central hyper-geometric distribution [10]. After achieving GO annotation for every unigene, the WEGO software (http://wego.genomics.org.cn/cgi-bin/wego/index.pl/) was used to perform GO classification and to construct a GO tree. The classification of the DEGs into the functional pathways was conducted using KEGG analysis. The KEGG automatic annotation server was used for KEGG Orthology (KO) and KEGG pathway annotation. Similarly, a BLASTX search against the COG database resulted in the classification of the unigenes into COG functional groups [34, 35]. The KOBAS [11] software was used to test the statistical enrichment of the DEGs in the KEGG pathways.

### TCGA analysis for the expression of DEGs in breast cancer patients

TCGA data portal was used to download clinical RNA-seq (IlluminaGA_RNASeqV2 platform) for breast cancer (BRCA) samples. For the RNA-seq data, the rsem.genes.normalized_results les (n = 22) were used without further normalization. The RNAseq data were grouped into Clinical Progressive Disease (PD) (n=4) and Complete Response (CR) (n=18) after treatment with TAM based on the TCGA annotation [29]. TCGA analyze_Filtering function was used to retain RNA transcript with mean which higher than threshold quantile=0.25 across all samples. Then TCGAanalyze_DEA function was applied to detect differentially expressed genes (DEGs) (log fold change >1.0 and FDR <-10e-2) in PD VS. CR.

### Protein-protein interaction network analysis

Protein-protein interaction network explore the down-stream relationship between proteins based on physical binding, genetic and functional relationship. We integrated DEGs(n=26) of patients and genes(n=98) involved in ER signal pathway using STRING website to explore the association between these DEGs with ER functional related genes.

### Gene expression by qRT-PCR

Finally, qRT-PCR was used to confirm our RNA-seq data. Two micrograms of total RNA was used to synthesize cDNA subsequently. Aliquots of 2 mg of mRNA were reverse-transcribed using a PrimeScript™ RT reagent Kit with the gDNA Eraser Kit according to the manufacturer’s instructions (TaKaRa Bio, China). SYBR Green-based qPCR was then performed on an ABI ViiA 7 Real-Time PCR system (Applied Biosystems, Foster City, CA, USA). *GAPDH* was used as the endogenous control, and all the reactions were performed in triplicate. Relative gene expression was calculated using the comparative cycle threshold (2^−ΔΔCT^) method. PCR cycling conditions consisted of 5 min at 95°C followed by 40 cycles of 15 s of denaturation at 95°C, 30 s of annealing at 55°C, and 30 s of extension at 72°C.

### Survival analysis

We downloaded the mRNA expression data from TCGA database and systematically evaluated the relationship of the DEGs and patients’ survival in over 400 breast cancer samples.

For single-gene survival analysis, the expression level of each gene in each sample was used to classify the samples according to the FPKM>1 or <1 as high expression or low expression groups. The feature genes with p-value < 0.01 were taken as potential genes association with TAM resistance. Survival curves were plotted from Kaplan-Meier estimates via the survival R package.

## Abbreviations

TAM: tamoxifen
DEGs: differentially expressed genes
TCGA: the cancer genome altas
GSEA: Gene Set Enrichment Analysis
TAMR/MCF-7: TAM-resistant cell line
PD: progressive disease
CR: complete response
